# Nutrition literacy and health-promoting lifestyle behaviors among university students: a cross-sectional study

**DOI:** 10.3389/fnut.2026.1863441

**Published:** 2026-07-07

**Authors:** Serap Balaban-Barta, Emre Duman

**Affiliations:** 1 Department of Nutrition and Dietetics, Faculty of Health Sciences, Gaziantep University, Gaziantep, Türkiye; 2 Department of Nutrition and Dietetics, Faculty of Health Sciences, Siirt University, Siirt, Türkiye

**Keywords:** dietary behaviors, health-promoting lifestyle behaviors, HPLP-II, nutrition literacy, university students, young adults

## Abstract

**Background:**

Nutrition literacy is considered an important determinant of healthy eating and health-promoting behaviors. This study aimed to examine the association between nutrition literacy and health-promoting lifestyle behaviors in university students.

**Methods:**

This cross-sectional study was conducted among university students aged 18–25 years at Gaziantep University, Türkiye. Data were collected through face-to-face interviews using a structured questionnaire that included sociodemographic characteristics, lifestyle habits, the Evaluation Instrument of Nutrition Literacy on Adults (EINLA), and the Health-Promoting Lifestyle Profile II (HPLP-II).

**Results:**

The final analysis included 400 university students. The mean age of the participants was 19.9 ± 1.7 years, and 88.0% were female. The mean total EINLA score was 27.90 ± 3.40, and the mean total HPLP-II score was 128.44 ± 19.01. Comparisons across nutrition literacy tertiles showed no statistically significant differences in total HPLP-II scores (*p* = 0.308). In multivariable linear regression analyses, total EINLA score was not significantly associated with either total HPLP-II score (β = −0.003, *p* = 0.949) or the HPLP-II nutrition subscale score (β = −0.083, *p* = 0.086). In the generalized additive model, however, the smooth term for EINLA showed a nonlinear association with the HPLP-II Nutrition subscale (*p* = 0.006). Regular physical activity and consuming ≥3 main meals per day were positively associated with both total HPLP-II score and the nutrition subscale score (*p* < 0.05 for all). In addition, students in the Natural Sciences had significantly lower total HPLP-II and nutrition subscale scores than those in the Health Sciences.

**Conclusion:**

In this sample of university students, nutrition literacy was not independently associated with overall health-promoting lifestyle behaviors after adjustment for covariates. Regular physical activity, main meal frequency, and academic department were associated with HPLP-II scores. These findings suggest that lifestyle behaviors in young adults should be interpreted in relation to both literacy-related and behavioral factors. Future studies may build on these findings by incorporating validated measures of digital food literacy and sustainable eating behaviors.

## 1 Introduction

University life is a vulnerable period during which young adults develop independent food choices, establish meal patterns and daily routine, and shape long-term lifestyle behaviors (1, 2). Lifestyle habits established during early adulthood often extend into later life and can substantially shape long-term health outcomes. Nevertheless, university students frequently encounter several challenges, such as irregular meal patterns, increased consumption of instant foods, sedentary behaviors, and stress, which may negatively affect their health (2, 3).

Nutrition literacy refers to the ability to obtain, understand, interpret, and use nutrition-related information in daily life (4). The instrument used in this study captures several domains of nutrition literacy, including general nutrition knowledge, reading comprehension, food groups, serving sizes, and food label reading and numeracy (5–7). These domains reflect both cognitive understanding and the practical application of nutrition information to everyday food choices (6, 8). Individuals with adequate nutrition literacy are expected to have the knowledge and skills necessary to understand serving sizes, prepare food, make healthy food choices, interpret food labels, and access reliable nutrition information (7). Students' dietary habits are frequently suboptimal and often do not align with healthy eating guidelines, which may have implications for weight status and chronic disease risk (9, 10). A study conducted among university students in Türkiye reported unfavorable dietary patterns, including frequent meal skipping and inadequate energy intake and concluded that students need greater nutrition-related knowledge regarding healthy eating habits (11). Similarly, a study conducted in Türkiye reported that university students had limited food and nutrition literacy and moderate healthy life skills, and that higher nutrition literacy was associated with more positive healthy life skills (12). Taken together, these findings underscore the potential importance of nutrition literacy as a modifiable factor that may influence dietary behaviors and broader health-promoting lifestyle patterns among university students.

In addition to nutrition literacy, healthy lifestyle behaviors constitute a broader framework that includes health responsibility, physical activity, stress management, interpersonal relations, spiritual growth, and healthy eating practices (13). Previous research has shown that health-promoting lifestyle behaviors among university students are influenced by sociodemographic and sociocultural factors, as well as educational characteristics such as academic major, age, education level, and financial status, and that these factors may play a significant role in overall health status (14). Given that lifestyle patterns develop across the lifespan, nutrition literacy may be a key modifiable factor in early adulthood, a period when dietary habits and broader health behaviors are actively shaped (15). At the same time, university students are increasingly exposed to nutrition-related content through social media and other online sources. However, existing nutrition literacy instruments may not fully reflect how students assess the credibility of such information within digital information environments (16). In contemporary food environments, digital nutrition literacy may be relevant to how students encounter and appraise nutrition information online (8). However, this construct was not directly measured in the present study. Although important studies have been conducted on nutrition literacy, the number of studies published in recent years remains limited. To our knowledge, few studies have examined the association between nutrition literacy and health-promoting lifestyle behaviors among university students using multidimensional assessment tools. Therefore, this study aimed to assess nutrition literacy and health-promoting lifestyle behaviors among university students and to examine the association between these constructs. The findings may also provide a baseline for future work on digital food literacy and sustainable eating behaviors in young adults.

## 2 Materials and Methods

### 2.1 Study design and participants

This cross-sectional study was conducted among university students aged 18–25 years. Participants were selected using convenience sampling from among eligible students. Recruitment was carried out between January and March 2026. Students were approached during class visits and in common student areas at Gaziantep University. The researchers first provided brief information about the purpose of the study, the voluntary nature of participation, and the approximate time required to complete the questionnaire. Students who expressed interest were then screened according to the eligibility criteria. Those who met the criteria and agreed to participate provided written informed consent before data collection. The questionnaire was administered face-to-face by the researchers in a setting that allowed participants to respond individually.

Students who were between 18 and 25 years of age, had sufficient cognitive ability to respond to the questionnaires and scales used in the study, had no diagnosis of cognitive impairment, and provided voluntary informed consent were included. Exclusion criteria were being younger than 18 or older than 25 years of age, inability to read and understand the language of the survey (Turkish), a history of cognitive impairment or psychiatric illness that could compromise the reliable completion of the study instruments, and incomplete or incorrectly completed questionnaire forms. A total of 423 students were initially recruited for the study. However, 23 students were excluded from the final analysis due to incomplete responses and multiple markings on the nutrition literacy questionnaire. Therefore, the final analysis was conducted with 400 students. The study was conducted in accordance with the Declaration of Helsinki and was approved by the Non-Interventional Clinical Research Ethics Committee of Gaziantep University before participant recruitment (approval number: 2026/41-778844; date of approval: January 7, 2026). Based on a previous study (17), an *a priori* power analysis was conducted in G^*^Power 3.1.9.7 for a planned multiple regression model with six primary predictors. Assuming an effect size of *f* ^2^ = 0.11, a significance level of 0.05, and 95% power, the minimum required sample size was estimated as 197 participants. In the final analyses, additional covariates were included to control for potential confounding. All participants were enrolled after eligibility screening and provision of written informed consent.

### 2.2 Data collection

Data were collected from university students through face-to-face interviews conducted by the researchers between January and March 2026. The study questionnaire consisted of three sections. The first section included items on participants' sociodemographic characteristics, such as age, sex, class year, living arrangement, physical activity habits, and dietary habits. Regular physical activity was recorded as a self-reported behavioral variable based on the questionnaire responses. The second section included the Evaluation Instrument of Nutrition Literacy on Adults (EINLA), which assesses general nutrition knowledge, reading comprehension, serving sizes, food groups, and food label reading/numeracy skills. The third section included the Health-Promoting Lifestyle Profile II (HPLP-II), which evaluates health-promoting behaviors across multiple lifestyle domains.

### 2.3 Anthropometric measurements

Body weight was measured using a Jawon X-CONTACT 356 (Jawon Medical Co. Ltd., Seoul, Republic of Korea), and height was measured using a wall-mounted stadiometer seca-220 (seca GmbH & Co. KG, Hamburg, Germany). Body weight was measured with participants wearing light clothing and no shoes. Height was measured without shoes, with participants standing upright and facing forward, with the heels together and the head positioned in the Frankfort horizontal plane. Body mass index (BMI) was calculated as weight in kilograms divided by height in meters squared (kg/m^2^). Participants were classified according to BMI as underweight (< 18.5 kg/m^2^), normal weight (18.5–24.99 kg/m^2^), overweight (25.0–29.99 kg/m^2^), or adults with obesity (≥30.0 kg/m^2^) (18). Measurements were performed and recorded by the same researcher to reduce inter-observer variation (19).

### 2.4 Evaluation instrument of nutrition literacy on adults (EINLA)

EINLA is a measurement tool developed to assess individuals' ability to access, understand, interpret, and apply basic nutrition-related information in daily life. Based on the integration of nutrition science and the concept of literacy, the instrument aims to evaluate the cognitive competencies underlying healthy eating behaviors. EINLA was originally developed and validated by Cesur et al. (6) for use in adult populations. During its development, existing instruments and the relevant literature on nutrition and health literacy were comprehensively reviewed in order to establish a valid and reliable tool suitable for use in Türkiye. The instrument was originally composed of 38 items and was reduced to 35 items following validity and reliability analyses. It comprises five domains: general nutrition knowledge (10 items), reading comprehension/interpretation (6 items), food groups (10 items), serving sizes (3 items), and label reading and numeracy (6 items). Each correct answer is scored as 1, while incorrect or missing answers are scored as 0, resulting in a total score ranging from 0 to 35. Based on the total score, nutrition literacy is categorized as inadequate (0–11), borderline (12–23), or adequate (24–35) (6). An English translation of the EINLA is provided for reader reference as Supplementary Table S1. However, since most participants in the present study fell into the adequate category, the original classification was not used in group comparisons; instead, tertiles based on the sample distribution were used. In the original validation study, Cronbach's alpha was reported as 0.75, whereas in the present study, the internal consistency coefficient for the total EINLA score was 0.67.

### 2.5 Health-promoting lifestyle profile II (HPLP-II)

Healthy lifestyle behaviors were assessed using the HPLP-II, originally developed by Susan Noble Walker et al. in 1987 (20) and revised in 1995 (21). The Turkish validity and reliability study was conducted by Bahar et al. (22). The scale consists of 52 items and includes six subscales: spiritual growth (9 items), health responsibility (9 items), physical activity (8 items), nutrition (9 items), interpersonal relations (9 items), and stress management (8 items). All items are positively worded and evaluate how frequently individuals engage in health-promoting behaviors. Responses are scored on a 4-point Likert scale ranging from 1 (never) to 4 (routinely), yielding a total score between 52 and 208. Higher scores indicate a greater adoption of health-promoting lifestyle behaviors (22). Cronbach's alpha for the original scale was reported as 0.94, while the Turkish adaptation reported a total scale Cronbach's alpha of 0.92. In the present study, Cronbach's alpha for the total HPLP-II score was 0.91.

### 2.6 Statistical analyses

All statistical analyses were conducted using IBM SPSS Statistics for Windows, version 26.0 (IBM Corp., NY, USA) and R software, version 4.5.2 (R Foundation for Statistical Computing, Vienna, Austria). Participants were divided into tertiles based on the distribution of total EINLA scores, using the 33rd and 66th percentiles. Accordingly, the T1 group included scores ranging from 5 to 27, the T2 group from 28 to 29, and the T3 group from 30 to 34. Descriptive group comparisons were performed across these tertile groups. In contrast, in the multivariable regression analyses, the total EINLA score was treated as a continuous variable and entered into the models as a standardized z-score. The distribution of continuous variables was assessed prior to analysis. Variables with an approximately normal distribution were presented as mean ± standard deviation (*x* ± SD), whereas skewed variables were summarized as median [Q1–Q3]. Categorical variables were expressed as frequency and percentages (%). Categorical variables were compared using the Pearson chi-square test, and exact tests were used when appropriate based on cell distribution. The normality of data was assessed by using the Kolmogorov-Smirnov test. One-way ANOVA was used to compare tertile groups for normally distributed variables, whereas the Kruskal–Wallis test was used for non-normally distributed variables. Effect sizes were reported, where appropriate, as partial eta-squared (η^2^p), epsilon-squared (ε^2^), and Cramer's V. Bivariate relationships among variables were examined using correlation analysis, and the results were visualized as a heatmap. Multivariable linear regression models were fitted to examine factors associated with the total HPLP-II score and the HPLP-II Nutrition subscale score. In addition to the standardized total EINLA score, the covariates included age, sex, BMI, regular physical activity, meal-skipping status, number of main meals per day, number of snacks per day, and academic department. The overall significance of the academic department was assessed using an omnibus test. The adjusted association between standardized total EINLA score and the HPLP-II Nutrition subscale score was further examined using a generalized additive model with a smooth term for EINLA and the same set of covariates. The effective degrees of freedom, test statistic, and *p*-value for the smooth term were reported to support interpretation of the modeled association. The reporting of this study followed the Strengthening the Reporting of Observational Studies in Epidemiology (STROBE) recommendations (23). A *p*-value of < 0.05 was considered statistically significant.

## 3 Results

Table 1 shows the overall distribution of participants' sociodemographic characteristics and lifestyle habits and their comparison across nutrition literacy tertiles. The mean age of the participants was 19.9 ± 1.7 years, and 88.0% were female. The median BMI was 20.9 [19.4–22.9] kg/m^2^, with most participants (74.9%) classified as normal weight. Comparisons across tertiles of nutrition literacy (T1, T2, and T3) showed no statistically significant differences in age, sex, height, body weight, BMI, or BMI category (*p* > 0.05). Likewise, regular physical activity, number of main meals and snacks, and meal-skipping habits did not differ significantly between tertile groups (*p* > 0.05). Academic department distribution was also similar across tertiles (*p* = 0.114). Furthermore, no statistically significant difference was observed in total HPLP-II scores across nutrition literacy tertiles (*p* = 0.308). Additional participant characteristics by EINLA tertiles are provided in Supplementary Table S2. Among these characteristics, eating out frequency and effort to increase nutrition knowledge differed significantly across EINLA tertiles (*p* = 0.011 and *p* = 0.014, respectively).

**Table 1:** Participant characteristics and HPLP-II total score overall and by nutrition literacy tertiles (EINLA).

Characteristic	Total (*n* = 400)	T1 (Low) (*n* = 143)	T2 (Mid) (*n* = 124)	T3 (High) (*n* = 133)	*p*-value	Effect size
**Age (years)**, *x ± SD*	19.9 ± 1.7	19.8 ± 1.7	20.1 ± 1.9	20.1 ± 1.6	0.446	*η2p* = 0.004
**Sex**, *n (%)*					0.647	*V* = 0.047
Female	352 (88.0)	123 (86.0)	111 (89.5)	118 (88.7)		
Male	48 (12.0)	20 (14.0)	13 (10.5)	15 (11.3)		
**Department**, *n (%)*					0.114	*V* = 0.096
Health sciences	258 (64.5)	87 (60.8)	76 (61.3)	95 (71.4)		
Natural sciences	51 (12.8)	18 (12.6)	22 (17.7)	11 (8.3)		
Social sciences	91 (22.8)	38 (26.6)	26 (21.0)	27 (20.3)		
**Weight (kg)**, *median [Q1–Q3]*	56.0, [52.00–63.0]	56.0, [50.0–63.0]	57.0, [52.0–63.8]	56.0, [52.00–64.5]	0.286	*ε^2^* = 0.001
**Height (cm)**, *x ± SD*	165.1 ± 7.2	165.0 ± 7.5	165.4 ± 6.9	164.9 ± 7.2	0.857	*η2p* = 0.001
**BMI (kg/m**^**2**^**)**, *median [Q1–Q3]*	20.9, [19.4–22.9]	20.8, [19.2–22.7]	20.9, [19.20–22.8]	21.1, [20.1–23.6]	0.179	*ε^2^* = 0.004
**Regular physical activity**, *n (%)*					0.452	*V* = 0.063
Yes	231 (57.8)	85 (59.4)	75 (60.5)	71 (53.4)		
No	169 (42.3)	58 (40.6)	49 (39.5)	62 (46.6)		
**Main meals/day**, *n (%)*					0.450	*V* = 0.063
< 3	204 (51.0)	75 (52.4)	67 (54.0)	62 (46.6)		
≥3	196 (49.0)	68 (47.6)	57 (46.0)	71 (53.4)		
**Snacks/day**, *n (%)*					0.269	*V* = 0.042
< 2	192 (48.0)	65 (45.5)	67 (54.0)	60 (45.1)		
≥2	208 (52.0)	78 (54.5)	57 (46.0)	73 (54.9)		
**Meal skipping**, *n (%)*					0.313	*V* = 0.076
Yes/Sometimes	348 (87.0)	126 (88.1)	111 (89.5)	111 (83.5)		
No	52 (13.0)	17 (11.9)	13 (10.5)	22 (16.5)		
**BMI category**, *n (%)*					0.512	*V* = 0.064
Underweight	56 (14.0)	20 (14.0)	19 (15.3)	17 (12.8)		
Normal	299 (74.9)	111 (77.6)	91 (73.4)	97 (72.9)		
Overweight/Obesity	45 (11.1)	12 (8.4)	14 (11.3)	19 (14.3)		
**Outcome variable**						
**HPLP–II Total Score**, *median [Q1–Q3]*	128.0, [116.0–140.0]	131.0, [114.0–140.0]	124.0, [115.3–138.5]	129.0, [116.5–140.5]	0.308	*ε^2^* < 0.001

EINLA tertiles were derived from the sample distribution: T1 = 5–27, T2 = 28–29, and T3 = 30–34. Values are presented as mean ± SD for approximately normally distributed continuous variables, median [Q1–Q3] for skewed continuous variables, and n (%) for categorical variables. Group differences were assessed using one-way ANOVA or Kruskal-Wallis tests for continuous variables and χ^2^ tests for categorical variables; exact tests were used when appropriate. Effect sizes are reported as partial eta-squared (ηp^2^), epsilon-squared (ε^2^), or Cramer's V, as appropriate.

Table 2 presents the descriptive statistics for the scales used in the study. The participants' mean total EINLA score was 27.90 ± 3.40 (median: 28.0). Among the EINLA subscales, Food Groups had the highest mean score (9.51 ± 1.02), whereas serving sizes had the lowest (1.72 ± 0.77). The mean total HPLP-II score was 128.44 ± 19.01 (median: 128.0). Within the HPLP-II subscales, the highest mean scores were observed for Spiritual Growth (26.43 ± 4.28) and Interpersonal Relations (25.67 ± 4.38), while Stress Management (19.15 ± 3.57) and Physical Activity (17.23 ± 4.81) had comparatively lower mean scores.

**Table 2:** Descriptive statistics of EINLA and HPLP-II total and subscale scores (N = 400).

Scale/Subscale	Items (k)	Possible score range	Mean ±SD	Median [Q1–Q3]
**EINLA total score**	35	0–35	27.90 ± 3.40	28.0 [26.0–30.0]
General nutrition knowledge	10	0–10	8.30 ± 1.65	9.0 [8.0–9.0]
Reading comprehension	6	0–6	5.10 ± 0.94	5.0 [5.0–6.0]
Food groups	10	0–10	9.51 ± 1.02	10.0 [9.0–10.0]
Serving sizes	3	0–3	1.72 ± 0.77	2.0 [1.0–2.0]
Label reading & numeracy	6	0–6	3.28 ± 1.24	3.0 [2.0–4.0]
**HPLP-II total score**	52	52–208	128.44 ± 19.01	128.0 [116.0–140.0]
Health responsibility	9	9–36	19.94 ± 4.69	20.0 [17.0–23.0]
Physical activity	8	8–32	17.23 ± 4.81	17.0 [14.0–20.0]
Nutrition	9	9–36	20.03 ± 4.06	20.0 [17.0–22.0]
Spiritual growth	9	9–36	26.43 ± 4.28	26.0 [23.3–29.8]
Interpersonal relations	9	9–36	25.67 ± 4.38	25.0 [23.0–29.0]
Stress management	8	8–32	19.15 ± 3.57	19.0 [17.0–21.0]

k: number of items. Values are x ± SD for approximately normal distributions and median [Q1–Q3] for skewed distributions.

Table 3 presents the multivariable linear regression models examining the factors associated with the total HPLP-II score and the HPLP-II Nutrition subscale score. The model for the total HPLP-II score was statistically significant (R^2^ = 0.089; adjusted R^2^ = 0.065; F(10, 389) = 3.778; *p* < 0.001). However, total EINLA score, demographic variables, and most dietary habit-related variables were not significantly associated with the total HPLP-II score (*p* > 0.05). Examination of the model coefficients showed that engaging in regular physical activity was positively associated with the total HPLP-II score (β = 0.198; B = 7.618; 95% CI: 3.895, 11.341; *p* < 0.001). Similarly, consuming ≥3 main meals per day was associated with a higher total HPLP-II score (β = 0.130; B = 4.921; 95% CI: 0.882, 8.961; *p* = 0.017). In addition, students in the Natural Sciences had lower total HPLP-II scores than those in the Health Sciences (β = −0.139; B = −7.937; 95% CI:−13.813,−2.060; *p* = 0.008). The model for the HPLP-II Nutrition subscale was also statistically significant (R^2^ = 0.118; adjusted R^2^ = 0.095; *F* (10, 389) = 5.197; *p* < 0.001), explaining 11.8% of the variance. In this model, total EINLA score showed a negative, although not statistically significant, association with the Nutrition subscale score (β = −0.083; B = −0.100; 95% CI:−0.214, 0.014; *p* = 0.086). Regular physical activity was positively associated with the Nutrition subscale score (β = 0.183; B = 1.503; 95% CI: 0.720, 2.285; *p* < 0.001), and consuming ≥3 main meals per day was likewise associated with higher Nutrition subscale scores (β = 0.145; B = 1.174; 95% CI: 0.325, 2.023; *p* = 0.007). In addition, students in the Natural Sciences had significantly lower Nutrition subscale scores than those in the Health Sciences (β = −0.177; B = −2.146; 95% CI:−3.381,−0.911; *p* = 0.001). No significant associations were observed for the remaining variables (*p* > 0.05). Multicollinearity was not a concern in the models, with all variance inflation factor (VIF) values below 1.3.

**Table 3:** Multivariable linear regression models for HPLP-II total score and HPLP-II nutrition subscale score.

Predictor	HPLP-II total			HPLP-II nutrition		
	β (sth.)	B [95% CI]	*p-value*	β (sth.)	B [95% CI]	*p-value*
EINLA total	−0.003	−0.018 [-0.560, 0.525]	0.949	−0.083	−0.100 [-0.214, 0.014]	0.086
Sex	0.029	1.685 [-4.100, 7.469]	0.567	0.026	0.323 [-0.892, 1.539]	0.602
Age	0.027	0.291 [-0.807, 1.388]	0.603	0.075	0.175 [-0.056, 0.406]	0.137
BMI	0.007	0.044 [-0.546, 0.634]	0.883	0.069	0.087 [-0.037, 0.211]	0.167
Regular physical activity	0.198	7.618 [3.895, 11.341]	**< 0.001**	0.183	1.503 [0.720, 2.285]	**< 0.001**
Meal skipping	−0.077	−4.325 [-10.205, 1.555]	0.149	−0.070	−0.841 [-2.076, 0.395]	0.182
Main meals/day	0.130	4.921 [0.882, 8.961]	**0.017**	0.145	1.174 [0.325, 2.023]	**0.007**
Snacks/day	0.002	0.077 [-3.664, 3.819]	0.968	0.022	0.178 [-0.608, 0.964]	0.656
**Department (omnibus test)** ^**†**^	—	—	**0.019**	—	—	**< 0.001**
Natural sciences vs Health sciences	−0.139	−7.937 [-13.813,−2.060]	**0.008**	−0.177	−2.146 [-3.381,−0.911]	**0.001**
Social sciences vs Health sciences	0.014	0.635 [-3.890, 5.161]	0.783	0.062	0.599 [-0.352, 1.550]	0.216
**Model fit statistics**						
**N**	400			400		
***R*** ^**2**^	0.089			0.118		
**Adjusted** ***R***^**2**^	0.065			0.095		
***F*** **(df1, df2)**	3.778 (10, 389)			5.197 (10, 389)		
**Model** ***p*****-value**	**< 0.001**			**< 0.001**		

Continuous predictors (EINLA total, age, and BMI) were standardized (z-scores) before modeling. Therefore, B coefficients for these predictors represent the change in the outcome (original units) per 1-SD increase in the predictor, whereas β coefficients are standardized regression coefficients. For categorical predictors, B coefficients represent differences relative to the reference category. Reference categories: female, no regular physical activity, no meal skipping, < 3 main meals/day, < 2 snacks/day, and health sciences for department. Department omnibus p-values are omnibus tests for the department indicators entered jointly. All VIFs < 1.3. The † symbol indicates the omnibus test for the department indicators entered jointly in the regression model. Bold p-values indicate statistically significant results at *p* < 0.05.

**Figure 1:**
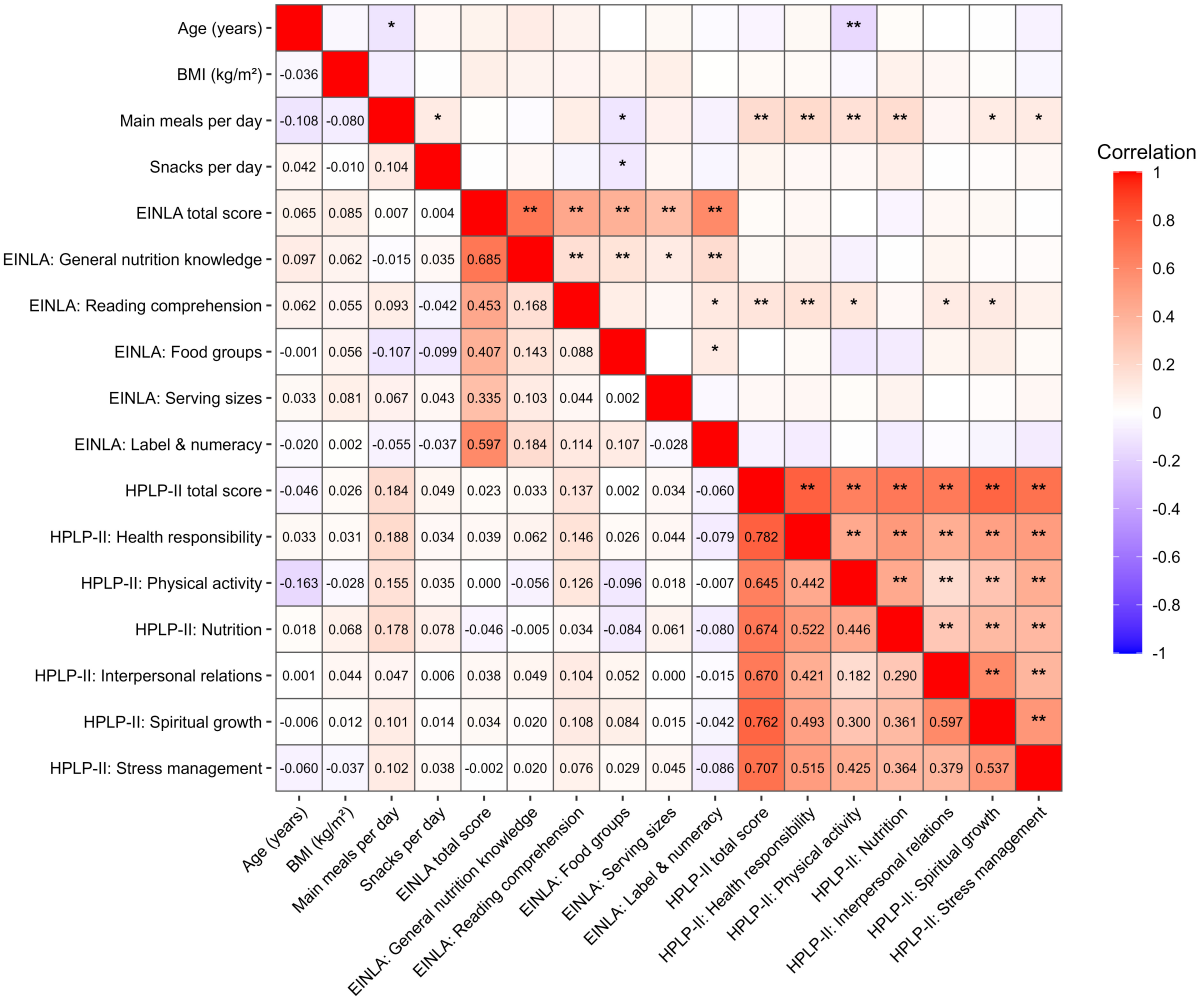
Correlation heatmap of EINLA, HPLP-II, and key covariates. Heatmap of bivariate correlations among age, BMI, meal frequency variables, EINLA total and domain scores, and HPLP-II total and subscale scores. Cells display correlation coefficients (lower triangle), with color intensity indicating magnitude and direction (red = positive; blue = negative). Asterisks indicate statistical significance (*p < 0.05; **p < 0.01).

Figure 1 shows the pairwise correlation coefficients and statistical significance levels among the study variables. Age was weakly but significantly negatively correlated with the number of main meals consumed per day (*r* = −0.108, *p* = 0.030) and with the HPLP-II Physical Activity subscale score (*r* = −0.163, *p* = 0.001). The number of main meals per day was positively correlated with both the total HPLP-II score (*r* = 0.184, *p* < 0.001) and the HPLP-II Nutrition subscale score (*r* = 0.178, *p* < 0.001). Additionally, the Spiritual Growth (*r* = 0.101, *p* = 0.043) and Stress Management (*r* = 0.102, *p* = 0.041) subscales showed weak yet statistically significant positive correlations. As expected, the total EINLA score showed positive correlations with its subscales, with the strongest associations observed for General Nutrition Knowledge (*r* = 0.685, *p* < 0.001) and Label Reading and Numeracy (*r* = 0.597, *p* < 0.001). In contrast, total EINLA score was not significantly correlated with either the total HPLP-II score (*r* = 0.023, *p* = 0.646) or the HPLP-II Nutrition subscale score (*r* = −0.046, *p* = 0.359).

**Figure 2:**
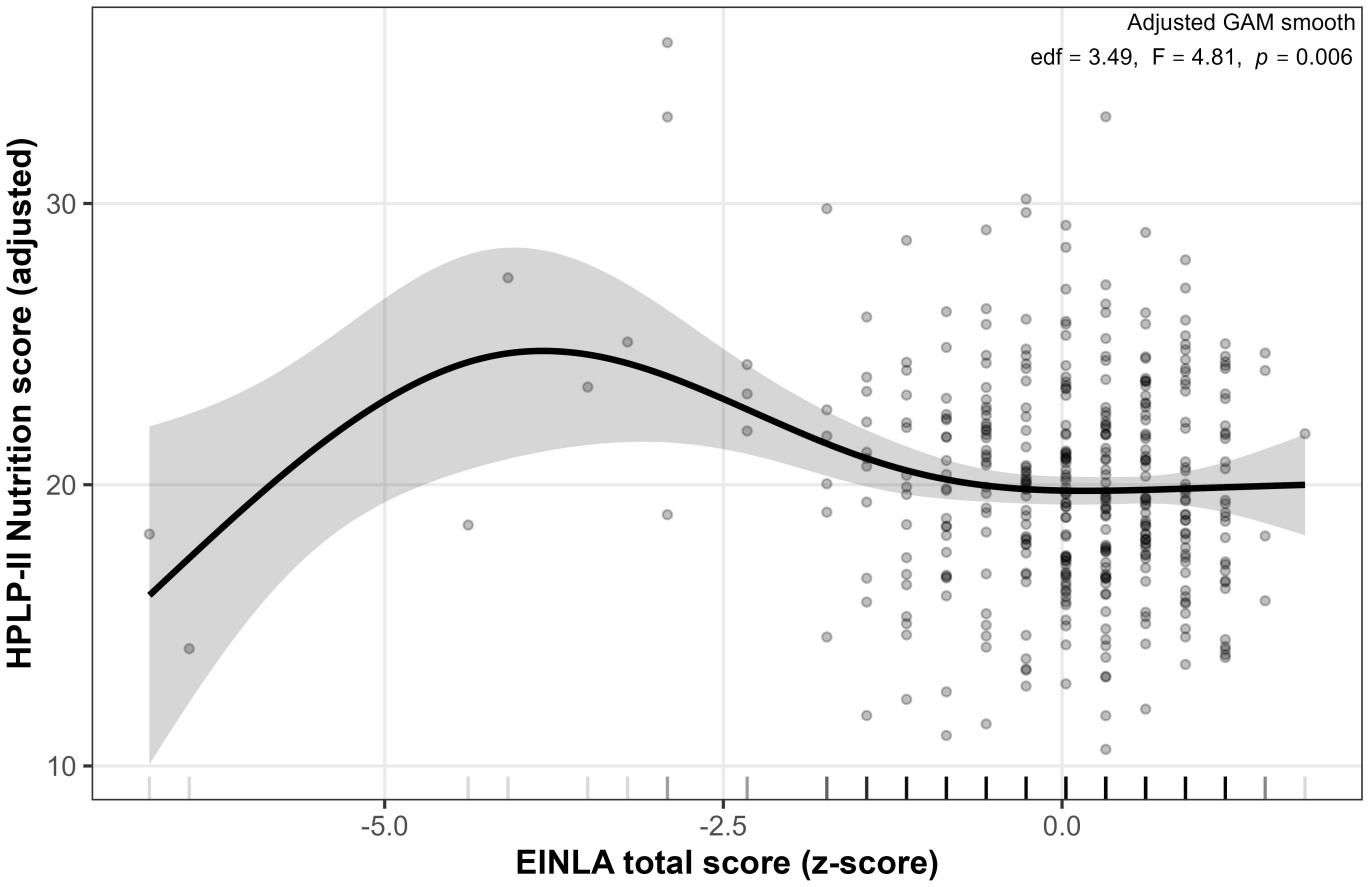
Adjusted association between EINLA total score and HPLP-II Nutrition subscale score. Generalized additive model plot showing the adjusted association between standardized EINLA total score and the HPLP-II Nutrition subscale score. The solid line represents the model-estimated association, and the shaded band indicates the 95% confidence interval. Points represent covariate-adjusted individual HPLP-II Nutrition scores. The model was adjusted for age, sex, BMI, regular physical activity, meal-skipping status, number of main meals per day, number of snacks per day, and academic department. The smooth term was statistically significant (edf = 3.49, *F* = 4.81, *p* = 0.006).

Figure 2 illustrates the adjusted association between the standardized total EINLA score and the HPLP-II Nutrition subscale. In the generalized additive model, the smooth term for EINLA was statistically significant, indicating a nonlinear association with the HPLP-II Nutrition subscale score (edf = 3.49, F = 4.81, *p* = 0.006). The model explained 15.4% of the deviance and had an adjusted R^2^ of 0.124. The confidence interval was wider at lower EINLA z-score values, which suggests greater uncertainty in this part of the distribution.

## 4 Discussion

In this cross-sectional study of university students, nutrition literacy was not independently associated with either overall health-promoting lifestyle behaviors or the nutrition subscale of the HPLP-II after adjustment for potential confounders. Although nutrition literacy is conceptually considered an important determinant of healthy eating and related behaviors, our findings suggest that its contribution to broader lifestyle patterns may be limited in this population. Regular physical activity and consuming at least three main meals per day were associated with higher total HPLP-II and Nutrition subscale scores. Students in the Natural Sciences had lower scores than those in the Health Sciences. These findings suggest that behavioral routines and educational context should be considered alongside nutrition literacy when interpreting health-promoting lifestyle behaviors in this sample.

The GAM analysis provided additional information beyond the linear regression model. Although total EINLA score was not linearly associated with the HPLP-II Nutrition subscale in the multivariable regression model, the smooth term in the GAM suggested a nonlinear adjusted pattern. This indicates that the association between EINLA and the Nutrition subscale did not follow a constant slope across the observed range of nutrition literacy. Because most participants had relatively high EINLA scores, the lower end of the distribution included fewer observations and wider confidence intervals. For this reason, the nonlinear pattern should be viewed as exploratory and should be confirmed in samples with greater variability in nutrition literacy. These findings support the interpretation that improvements in nutrition literacy alone may not be sufficient to produce direct behavioral change and may reflect a knowledge–behavior gap in this population. The absence of an association between nutrition literacy and HPLP-II scores may partly reflect the scope of the literacy instrument. EINLA primarily assesses nutrition literacy domains, such as basic nutrition knowledge, comprehension, food groups, and portion-related understanding. However, university students are increasingly exposed to nutrition-related information through digital platforms, and existing instruments may not fully capture their ability to evaluate the credibility, relevance, and applicability of such information in online environments (8, 24). For example, in a study conducted among students in Vietnam, digital healthy diet literacy was measured as a construct distinct from general health literacy, and higher digital healthy diet literacy scores were associated with healthier eating behavior. This finding suggests that digital nutrition literacy may represent an additional domain of competence that is not fully captured by conventional nutrition literacy measures (25). Consistent with this view, a previous study among university students found that dietary habits were driven more strongly by attitudes than by knowledge alone (26). In the same study, participants who received nutrition education demonstrated higher knowledge levels on days 3 and 100. However, a statistically significant difference in attitudes was observed only on day 100, whereas no marked difference was found between the groups with respect to the translation of knowledge into practice (26). Furthermore, in another assessment of diet quality, a marked improvement was observed in the intervention group on day 3, although this improvement was found to decrease by day 100 (26). Likewise, in another study that accounted for multiple individual and environmental variables, nutrition literacy was identified as a predictor of healthy eating behavior. Moreover, nutrition literacy has been suggested to mediate the relationship between various external influences and healthy eating behavior (27). Taken together, previous studies suggest that eating behavior is related not only to nutrition literacy but also to attitudes, social support, the university food environment, and students' ability to navigate nutrition information in digital settings (26–28). In the present study, however, digital nutrition literacy was not directly measured. Therefore, this interpretation should be considered contextual rather than confirmatory.

As shown in Table 2, students scored highest in the Food Groups domain and lowest in Portion Sizes. This pattern suggests that recognizing healthy food categories may be easier for students than applying this knowledge to estimate appropriate intake amounts in daily life. In the HPLP-II, Physical Activity and Stress Management were among the lowest-scoring domains, indicating that these behaviorally demanding aspects of a healthy lifestyle may be more difficult to maintain during university life. Previous studies have reported similar findings, suggesting that the high stress and intensive academic demands associated with university life may negatively influence physical activity and stress management (29, 30).

On the other hand, current literature also includes studies reporting that nutrition knowledge or nutrition literacy is associated with healthier dietary patterns and more favorable eating behaviors (31–33). For instance, a study among female university students in Türkiye found that higher nutrition knowledge was associated with increased intake of water, fruits and vegetables, grains, and dietary fiber, while total carbohydrate intake was reported to be lower (32). Another study found that nursing students had high levels of nutrition knowledge, although their practical application skills remained low. In the same study, nutrition literacy was reported to explain 44% of the variation in eating behaviors (33). Taken together, these studies suggest that nutrition-related knowledge may be linked to behavior. However, a comparable direct association was not observed in the present study. Possible explanations for this discrepancy include differences in sample characteristics, measurement instruments, and the specific variables examined (32, 33). Overall, the relationship between nutrition literacy and behavior may vary across different populations and contexts. This association is likely to be influenced by additional factors, particularly practical skills, motivation, and environmental opportunities (26–28).

Regular physical activity and consuming at least three main meals per day were the behavioral factors most consistently associated with HPLP-II scores. This pattern is consistent with studies suggesting that physical activity and dietary behaviors tend to be linked among university students (34, 35). In a large sample of university students, Lonati et al. (35) reported a positive association between physical activity and a healthy diet, with students reporting more physical activity also showing more adequate dietary patterns. Similarly, Doak et al. (34) reported associations between dietary quality and lifestyle behaviors among higher education students. In the present study, students who reported regular physical activity may also have had more structured daily routines, which could partly explain their higher Nutrition subscale scores. In this context, regular physical activity may reflect not only exercise behavior itself, but also a broader pattern of health awareness, time management, motivation, and self-care. Likewise, consuming three or more main meals per day may represent a more structured daily routine, which could facilitate healthier nutrition-related behaviors. The lower HPLP-II scores observed among students in the Natural Sciences, compared with those in the Health Sciences, may reflect differences in exposure to health-related coursework or applied health messages. This interpretation is supported by previous evidence showing higher overall HPLP and nutrition scores among health-related students than non-health-related students (36). However, curricular exposure was not directly measured in the present study, and this explanation should therefore be treated cautiously. Overall, these findings suggest that nutrition literacy should be interpreted within a broader behavioral and educational context, rather than as an isolated predictor of lifestyle behavior.

### 4.1 Limitations and future directions

Several limitations should be considered when interpreting these findings. The cross-sectional design limits causal inference, and the results describe associations observed at one point in time. The single-center setting, the age range of 18–25 years, and the predominance of female and health sciences students may limit generalizability. Some behavioral variables, including physical activity and meal patterns, were self-reported and may have been affected by recall or social desirability bias. Although the regression models included several covariates, unmeasured factors such as income, housing conditions, food access, sleep, psychological status, and motivation for healthy eating may still have influenced the findings. The distribution of EINLA scores was also narrow, with most participants falling in the adequate range. This may have reduced variability and limited the ability to detect differences across nutrition literacy levels. In addition, the internal consistency of the EINLA was lower than that of the HPLP-II in this sample. This may have increased measurement error and attenuated the observed associations between nutrition literacy and HPLP-II outcomes.

The present study did not measure digital food literacy, exposure to online nutrition information, or sustainable eating behaviors. The results should therefore be read as evidence on conventional nutrition literacy and health-promoting lifestyle behaviors. Future studies could extend this work by using validated digital food literacy instruments and by including dietary indicators that capture sustainability-related practices.

## 5 Conclusion

In this cross-sectional study, nutrition literacy was not independently associated with overall HPLP-II scores after adjustment for covariates. Regular physical activity, consuming three or more main meals per day, and academic department were associated with HPLP-II outcomes. These findings suggest that health-promoting behaviors among university students should be interpreted in relation to daily routines and educational context as well as literacy-related factors. University health-promotion strategies may benefit from combining nutrition literacy components with behavioral approaches that support physical activity and regular meal patterns. Because digital food literacy and sustainable eating behaviors were not assessed, future studies should examine these constructs directly using validated measures and longitudinal designs.

## Data Availability

The original contributions presented in the study are included in the article/Supplementary material, further inquiries can be directed to the corresponding author.
