# Supplementary material for: Nutrition literacy and health-promoting lifestyle behaviors among university students: a cross-sectional study
Source: Front Nutr. 2026 Jul 7;13:1863441. doi: 10.3389/fnut.2026.1863441 (PMC13385125; doi:10.3389/fnut.2026.1863441)
Supplement: Supplementary file 1 [file Table_1.pdf]

Supplementary Table S1

Evaluation Instrument of Nutrition Literacy on Adults (EINLA)

Translated from the original Turkish instrument for reader reference.

Note. *This English version is intended to facilitate interpretation by international readers. For formal cross-cultural use, psychometric validation of the translated version would be required.*

Section 1. General Nutrition Knowledge

**1. Which of the following grain products is the most beneficial for health?**			
a. Pasta	b. Rice	c. Corn flour	d. Whole wheat bread
**2. Which is the healthiest source of fat?**			
a. Margarine	b. Tallow	c. Corn oil	d. Olive oil
**3. Which is necessary for dental health?**			
a. Iron	b. Iodine	c. Sodium	d. Fluoride
**4. Which of the following is not a food high in salt?**			
a. Sausage	b. Pickle	c. Olives	d. Fresh peas
**5. .... is necessary for bone health.**			
a. Calcium	b. Magnesium	c. Potassium	d. Iodine
**6. Adults should drink .... ……….water every day.**			
a. One to two glasses	b. Three to four glasses	c. Eight to ten glasses	d. Whenever they feel thirsty
**7. Consuming beverages such as .... with meals reduces your body's ability to use iron.**			
a. Orange juice	b. Lemonade	c. Linden tea	d. Black tea
**8.……......... helps protect against illnesses such as flu and the common cold and supports healthier gums.**			
a. Vitamin C	b. Vitamin B	c. Vitamin A	d. Vitamin D
**For Questions 9 and 10: A woman buys fish, bread, milk in a carton, canned food, eggs, and tomatoes at the supermarket. She then completes some other errands and returns home two and a half hours later. As soon as she gets home, she places the milk and frozen fish in the refrigerator.**			
9**. Which of the foods purchased by her is most likely to spoil first?**			
a. Fish	b. Milk	c. Tomatoes	d. Eggs
**10. At the latest, within how many hours should the fish be placed in the refrigerator?**			
a. 2 hours	b. 3 hours	c. 4 hours	d. 5 hours

Section 2. Reading Comprehension and Interpretation

**From birth onward, we obtain all the substances our bodies need for growth and development and for a healthy, long life through food. Consuming foods with similar nutritional content at every meal may result in a monotonous and nutritionally unbalanced diet, which is not considered healthy. Healthy eating requires consuming adequate amounts from a variety of food groups each day, including vegetables, fruits, meat, milk, and grain products, while limiting the intake of foods high in saturated fat, trans fat, cholesterol, salt, and sugar. When any food group is omitted, consumed in inadequate or excessive amounts, or when foods high in fat, cholesterol, salt, or sugar are consumed excessively, growth and development may be impaired, and health may be adversely affected. Today, with changes in eating habits and increasingly sedentary lifestyles, dietary behaviors play an important role in the development of health problems such as cardiovascular diseases, many types of cancer, anemia, hypertension, diabetes, osteoporosis, and obesity. To avoid adverse effects on health, it is also important that foods be fresh and clean. Therefore, before purchase, consumers should examine label information such as the production date, expiration date, and official authorization statement.**			
**1. For healthy eating, foods such as meat and milk should be consumed ....**			
a. In excess	b. In sufficient amounts	c. In small amounts	d. Rarely
**2. In individuals with unhealthy dietary habits, diseases such as .... may develop.**			
a. AIDS	b. Hepatitis B	c. Hypertension	d. Measles
**3. Some foods, such as ...., should be consumed in limited amounts for healthy eating.**			
a. Vegetables	b. Salt	c. Milk	d. Grain product
**4. If we consume foods with .... nutrient content at every meal, we will have a healthy diet.**			
a. The same	b. Various	c. Similar	d. Small amount of
**5. If you were asked to choose a healthy food, which of the foods shown in the photographs would you prefer?**			
a. Hamburger menu 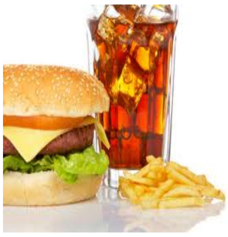	b. Salad and fish 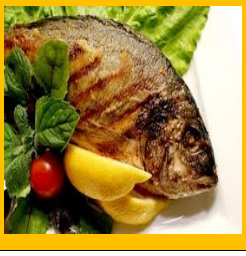	c. French fries 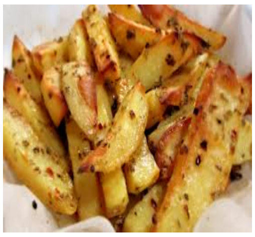	d. Pasta with tomato sauce 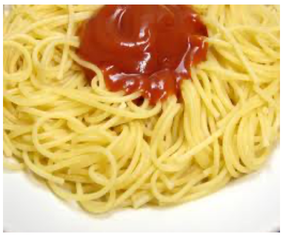
**6. What would you do if you noticed that the product you purchased had passed its expiration date?**			
a. I would use it if the expiration date had not passed by very much.	b. I would use it if there were no color change, unpleasant odor, etc.	c. I would return it and warn the seller	d. I would not use it; I would throw it away

Section 3. Food Groups

Write the letters shown on the foods in the pictures into the appropriate food-group categories in the figure.

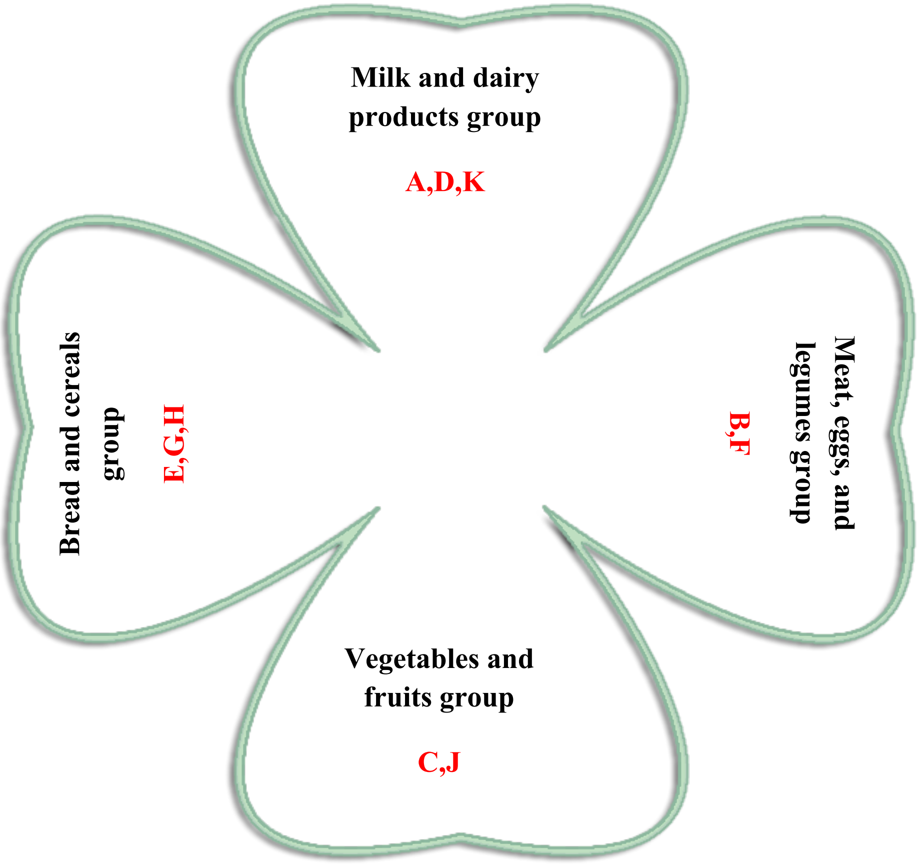

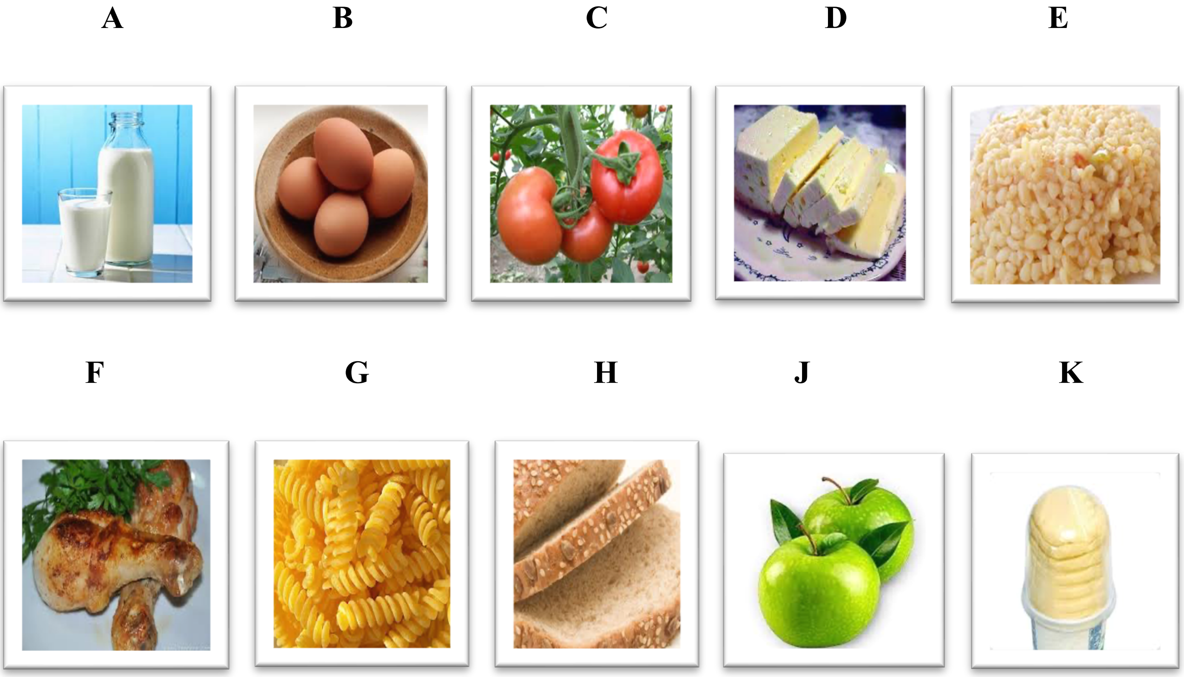

Section 4. Serving Sizes

Note: The amount for one serving of each food is indicated in the boxes next to the questions.

**1. Foods from the milk group should be consumed .... per day.**<br>**One serving: milk = one glass (200 g);**<br>**cheese = two matchbox-sized pieces (60 g)**			
a. One serving	b. Two servings	c. Four servings	d. Five servings
**2. Foods from the meat, eggs, and legumes group should be consumed .... per day.**<br>**One serving: legumes = one small glass (90 g);**<br>**meat, chicken, fish, etc. = 50–60 g (about two grilled meatballs);**<br>**eggs = 2 eggs**			
a. One serving	b. Two servings	c. Four servings	d. Five servings
**3. For a healthy diet, ... nuts should be consumed each day.**<br>**Walnuts, hazelnuts, almonds, etc.: one handful (30 g)**			
a. None	b. One handful	c. Two handfuls	d. Three handfuls

Section 5. Label Reading and Numeracy

Body Mass Index (BMI): $\frac{\textbf{Weight (kg)}}{\textbf{height squared (m}^2\textbf{)}}$

BMI categories:

a. Underweight: <20 b. Normal: 20.0–24.9 c. Overweight: 25.0–29.9 d. Obese: ≥30.0

**1. Your BMI : …………………**
**2. Your BMI category evaluation:……………**
Ingredients: wheat flour, vegetable oil, glucose syrup, flavoring, salt, sugar, whey powder, tomato paste, potato flour, raising agents (sodium and ammonium hydrogen carbonate).

Batch/lot no: 100003335-5444 Place of production: Sivas Made in Turkey Net weight: 90 g

Produced with the permission of the Ministry of Food, Agriculture and Livestock dated 2013 and numbered 10002.

Nutrition Facts	100 g	1 package (90 g)
Energy (kcal)	456	410
Protein (g)	7.2	6.5
Carbohydrate (g)	63.3	57.0
Fat (g)	19.3	17.3
Sodium (mg)	907	816

3. If you consume three packages of this product, how many kilocalories would you take in?

a. 1230 kcal	b. 1368 kcal	c.410 kcal	d. 820 kcal

4. How many kilocalories are provided by the fat in 100 grams of this product? (1 g fat = 9 kcal)

a. 36.6 kcal	b.155.7 kcal	c. 456 kcal	d. 173.7 kcal

5. People with which condition should consume this food with caution or avoid excessive intake?

a. Anemia	b. Hypertension	c. Osteoporosis	d. Osteoporosis

6. Which of the following items of information required on a food label is missing from the food label above?

a. Authorization from the Ministry of Food, Agriculture and Livestock	b. Logo of the Turkish Standards Institution	c. Expiration date	d. Country where the food was produced

Reference: Cesur B, Koçoğlu G, Sümer H. Evaluation instrument of nutrition literacy on adults (EINLA) a validity and reliability study. Integr Food Nutr Metab 2015;2:127-30. Doi: 10.15761/IFNM.1000114
