# Supplementary material for: Nutrition literacy and health-promoting lifestyle behaviors among university students: a cross-sectional study
Source: Front Nutr. 2026 Jul 7;13:1863441. doi: 10.3389/fnut.2026.1863441 (PMC13385125; doi:10.3389/fnut.2026.1863441)
Supplement: Supplementary file 2 [file Table_2.docx]

**Supplementary Table S2.** Extended participant characteristics by EINLA tertiles

| **Characteristic** | **T1 (Low)**  **(n = 143)** | **T2 (Mid)**  **(n = 124)** | **T3 (High)**  **(n = 133)** | **Total**  **(n = 400)** | ***p*-value** | **Cramer's V** |
| --- | --- | --- | --- | --- | --- | --- |
| **Academic year** |  |  |  |  | 0.238 | 0.100 |
| 1st year | 82 (57.3) | 66 (53.2) | 60 (45.1) | 208 (52.0) |  |  |
| 2nd year | 16 (11.2) | 14 (11.3) | 18 (13.6) | 48 (12.0) |  |  |
| 3rd year | 16 (11.2) | 13 (10.5) | 26 (19.5) | 55 (13.7) |  |  |
| 4th year or higher | 29 (20.3) | 31 (25.0) | 29 (21.8) | 89 (22.3) |  |  |
| **Residence** |  |  |  |  | 0.421 | 0.070 |
| With family | 30 (21.0) | 31 (25.0) | 40 (30.1) | 101 (25.3) |  |  |
| Dormitory | 101 (70.6) | 86 (69.4) | 86 (64.6) | 273 (68.2) |  |  |
| House | 12 (8.4) | 7 (5.6) | 7 (5.3) | 26 (6.5) |  |  |
| **Number of siblings** |  |  |  |  | 0.738 | 0.081 |
| 0 | 6 (4.3) | 4 (3.3) | 5 (3.8) | 15 (3.8) |  |  |
| 1 | 40 (28.4) | 40 (32.5) | 40 (30.1) | 120 (30.2) |  |  |
| 2 | 48 (34.0) | 44 (35.8) | 51 (38.3) | 143 (36.0) |  |  |
| 3 | 23 (16.3) | 24 (19.5) | 19 (14.3) | 66 (16.6) |  |  |
| 4+ | 26 (17.0) | 12 (8.9) | 18 (13.5) | 56 (13.4) |  |  |
| **Eating out frequency** |  |  |  |  | **0.011** | 0.170 |
| Every meal | 5 (3.5) | 1 (0.8) | 1 (0.8) | 7 (1.8) |  |  |
| Every day | 18 (12.6) | 11 (8.9) | 9 (6.8) | 38 (9.5) |  |  |
| 1–2×/week | 47 (32.8) | 58 (46.7) | 65 (48.9) | 170 (42.5) |  |  |
| 2–3×/week | 43 (30.1) | 27 (21.8) | 22 (16.5) | 92 (23.0) |  |  |
| 4–5×/week | 16 (11.2) | 12 (9.7) | 10 (7.5) | 38 (9.5) |  |  |
| Once a month | 14 (9.8) | 15 (12.1) | 26 (19.5) | 55 (13.7) |  |  |
| **Effort to increase nutrition knowledge** |  |  |  |  | **0.014** | 0.146 |
| No | 60 (42.0) | 46 (37.1) | 34 (25.6) | 140 (35.0) |  |  |
| Yes | 83 (58.0) | 78 (62.9) | 99 (74.4) | 260 (65.0) |  |  |
| **Primary source of nutrition information** |  |  |  |  | 0.526 | 0.106 |
| Physician | 5 (3.5) | 3 (2.4) | 5 (3.8) | 13 (3.3) |  |  |
| Dietitian | 21 (14.7) | 17 (13.7) | 18 (13.5) | 56 (14.0) |  |  |
| TV/Internet | 77 (53.8) | 74 (59.7) | 79 (59.4) | 230 (57.5) |  |  |
| Friends | 25 (17.5) | 13 (10.5) | 10 (7.5) | 48 (12.0) |  |  |
| Magazine etc. | 9 (6.3) | 12 (9.7) | 15 (11.3) | 36 (9.0) |  |  |
| Family | 6 (4.2) | 5 (4.0) | 6 (4.5) | 17 (4.2) |  |  |

*Note: Values are n (%) with column percentages calculated within each EINLA tertile. p-values are from Pearson’s chi-square tests; effect sizes are Cramer's V. To limit sparse cells, academic year was collapsed as 4th year or higher, and siblings as 4+.*
